# Chickpea (*Cicer arietinum* L.) Protein Isolate: Amino Acid Quality and Enhanced HDL Functionality in High-Fat-Diet-Fed Rats

**DOI:** 10.3390/foods15203765

**Published:** 2026-10-21

**Authors:** Hakima Mir, Djamil Krouf, Fatima Zohra Alachaher, Akila Guenzet, Hadj Mostefa Khelladi, Sadia Berzou, Imane Hammana

**Affiliations:** 1 Laboratory of Beneficial Microorganisms, Functional Food and Health, Faculty of Nature and Life Sciences, Abdelhamid Ibn Badis University, Route Nationale N 11, Kharouba, Mostaganem 27000, Algeria; fatima.alachaher@univ-mosta.dz; 2 Laboratory of Clinical and Metabolic Nutrition, Faculty of Nature and Life Sciences, University Oran 1 Ahmed Ben Bella, Oran 31000, Algeria; lncmkrouf@gmail.com (D.K.); guenzeta@gmail.com (A.G.); khmbiologie@gmail.com (H.M.K.); sadberzou2024@gmail.com (S.B.); 3 Health Technology Assessment Unit, Centre Hospitalier de l’Université de Montréal, Montreal, QC H2X 0A9, Canada; ihammana@gmail.com

**Keywords:** *Cicer arietinum*, chickpea protein isolate, food ingredient, plant protein, protein quality, dyslipidemia, hepatic lipid accumulation, HDL functionality, paraoxonase-1, oxidative stress

## Abstract

Chickpea (*Cicer arietinum* L.) is one of the most widely consumed grain legumes worldwide, and its protein isolates are increasingly used as ingredients in plant-based foods. This study assessed the nutritional quality of a chickpea protein isolate produced from locally grown seeds by alkaline solubilization and isoelectric precipitation and evaluated it in vivo against casein under a high-fat dietary challenge. In total, 30 male Wistar rats were allocated to three weight-matched groups for 28 days: control (20% casein), high-fat diet (HFD, 20% casein plus 20% sheep fat), and HFD with casein replaced by 20% chickpea protein isolate (HFD-CP). No indispensable amino acid determined in the isolate (tryptophan was not analyzed) fell below the FAO/WHO reference pattern. Compared with HFD, HFD-CP lowered body weight gain, adiposity, serum leptin (−29%), total cholesterol (−23%), LDL-cholesterol (−36%), hepatic triglycerides (−26%), hepatic cholesterol (−29%), and serum and hepatic thiobarbituric acid reactive substances (−29% and −35%) and increased superoxide dismutase, catalase, lecithin-cholesterol acyltransferase (+37%), and paraoxonase-1 (+33%) activities (*p* < 0.05), with no significant difference in energy intake between the high-fat groups. As an ingredient, the isolate therefore retains a high nutritional quality when substituted for an animal protein, supporting its use in the formulation of plant-based foods, although the present design does not allow the observed effects to be assigned to its protein fraction alone.

## 1. Introduction

Chickpea (*Cicer arietinum* L.) is one of the most widely grown and consumed grain legumes worldwide and a structural component of Mediterranean and North African cropping systems and dietary patterns [1]. As a cool-season pulse, it combines modest input requirements with a seed protein content that is high by cereal standards, which makes it a strategic crop in regions where the expansion of animal protein production is constrained by land and water availability. Chickpea is therefore an attractive raw material for the plant protein ingredients that the food industry is currently seeking.

Fractionation is one of the most direct routes to that added value. Protein isolates and concentrates obtained from pulse flours are increasingly used as ingredients in bakery, beverage, and meat-analogue formulations, and chickpea protein has attracted particular attention for its solubility, emulsifying, and gelling behaviour [2,3]. Alkaline solubilization followed by isoelectric precipitation remains the most accessible preparation route at pilot scale and is well documented for pea, chickpea, and lentil [4]. Interest in these ingredients is reinforced by the nutritional and environmental arguments supporting a partial shift from animal to plant protein sources [5].

Technofunctionality alone, however, does not establish the quality of a fractionated ingredient. The nutritional quality of the protein fraction, meaning its amino acid profile, its digestibility, and its physiological effects once consumed, is an equally important quality attribute of the ingredient and one that is far less frequently characterized [6,7]. Chickpea protein is rich in arginine and displays a low lysine-to-arginine ratio, a feature associated with hypocholesterolemic and cardioprotective effects [7,8], and chickpea-derived peptides show antioxidant properties [9]. At the dietary pattern level, plant-based diets and the substitution of pulses for animal-protein foods have been associated with improved cardiometabolic outcomes and lipid profiles [10,11,12]. Most of this evidence nevertheless concerns whole seeds or crude flours, in which the contribution of the protein fraction cannot be separated from that of fibre, starch, and phytochemicals.

A demanding way to test the nutritional quality of a protein ingredient is to substitute it for a reference animal protein under a dietary challenge. High-fat feeding drives central obesity, dyslipidemia, impaired glucose regulation, and chronic low-grade inflammation [13,14,15], and non-alcoholic fatty liver disease (NAFLD) has become the hepatic manifestation of this dysfunction [16]. Hepatic lipid accumulation reflects an imbalance between fatty acid uptake, de novo lipogenesis, and fatty acid oxidation [17,18,19] and is accompanied by oxidative stress that amplifies lipid peroxidation and tissue damage [20].

Beyond circulating lipoprotein concentrations, the functionality of high-density lipoprotein (HDL) particles depends on two associated enzymes: lecithin-cholesterol acyltransferase (LCAT), which esterifies free cholesterol and drives HDL maturation and reverse cholesterol transport, and paraoxonase-1 (PON-1), which protects lipoproteins against oxidative modification and preserves HDL antioxidant capacity [21,22]. High-fat feeding impairs both enzymes and reduces HDL functionality independently of HDL-cholesterol concentration, so dietary ingredients able to preserve them are of particular interest.

The present study, therefore, evaluated a chickpea protein isolate prepared from locally produced seeds, first through chemical and amino acid characterization, and then by substituting it for casein in a high-fat diet fed to rats for 28 days. Body weight and adiposity, serum leptin, serum and hepatic lipid profiles, oxidative stress markers, and HDL-associated enzyme activities were measured. We hypothesized that replacing casein with chickpea protein isolate would attenuate hepatic lipid accumulation and dyslipidemia, reduce oxidative stress, and enhance HDL functionality and that the isolated protein fraction would therefore constitute a nutritionally valuable ingredient in its own right.

## 2. Materials and Methods

### 2.1. Chickpea Protein Isolation

Dried chickpea (*Cicer arietinum* L.) seeds (local Kabuli-type landrace, cultivated in the Mascara region, northwestern Algeria, harvested in June 2024) were obtained from a certified local supplier (Oran, Algeria), with provenance and harvest year certified by the supplier, and were cleaned and milled into fine flour. Chickpea protein isolate was prepared by alkaline solubilization and isoelectric precipitation adapted from Boye et al. [4]. Flour was suspended in cold distilled water (1:10, *w*/*v*), the pH was adjusted to 9.0 with 2 N NaOH, and the suspension was stirred for 2 h at room temperature. After centrifugation at 4400 rpm (approximately 3000× *g*) for 20 min at 4 °C (Centrifuge 5702, Eppendorf, Hamburg, Germany), proteins in the supernatant were precipitated at pH 4.5 with 2 N HCl and recovered by centrifugation under the same conditions. The curd was washed with cold distilled water to remove residual soluble components and then neutralized to pH 7.0, freeze-dried, and ground. The curd-to-water ratio, the duration and number of washing cycles, and the sieve aperture were not retained in the contemporaneous laboratory record and are therefore not reported. Protein content was determined by the Kjeldahl method [23].

### 2.2. Chemical and Amino Acid Composition

Analyses were performed in triplicate. Crude protein was determined by the Kjeldahl method [23] and crude lipid by chloroform-methanol extraction according to Folch et al. [24]. Moisture and ash were determined according to AOAC official methods [25], as weight loss after drying at 105 °C for 24 h and incineration at 550 °C for 6 h, respectively. Amino acid composition was analyzed by high-performance liquid chromatography after acid hydrolysis (6 N HCl, 110 °C, 24 h, under nitrogen) using pre-column derivatization as described by Bidlingmeyer et al. [26]; sulfur-containing amino acids were determined after performic acid oxidation. Amino acids were expressed as g/100 g protein. Protein quality indices were derived from the amino acid composition. For each indispensable amino acid, the amino acid ratio was calculated as its content in the test protein divided by its content in the FAO/WHO reference patterns for the older child, adolescent, and adult and for the child aged 6 months to 3 years [27]. The lowest ratio was taken as the amino acid score and identified the first limiting amino acid, and the geometric mean of the individual ratios was used as an essential amino acid index. Because tryptophan is destroyed by acid hydrolysis and was not determined, all indices were computed on eight of the nine indispensable amino acids, and they were not corrected for digestibility and therefore constitute neither protein digestibility corrected amino acid score (PDCAAS) nor digestible indispensable amino acid score (DIAAS) values. Dietary sulfur amino acid supply was calculated from the amino acid composition of the protein sources and expressed as methionine equivalents, 1.24 g cystine being considered equivalent to 1.0 g methionine. The fatty acid composition of the sheep fat batch used in the experimental diets (SF-2024-06) was determined in triplicate (*n* = 3) by gas chromatography with flame ionization detection. Fat samples were dissolved directly in *n*-hexane, and fatty acid methyl esters were prepared by transesterification with 2 M methanolic potassium hydroxide at room temperature. Methyl esters were separated on a Shimadzu GC-2010 Plus gas chromatograph (Shimadzu, Kyoto, Japan) fitted with a flame ionization detector and a CP-Sil 88 capillary column (100 m × 0.25 mm i.d., 0.20 µm film thickness; Agilent Technologies, Santa Clara, CA, USA), with high-purity helium as carrier gas at a constant linear velocity of 20 cm/s and a split ratio of 1:50. Injector and detector temperatures were 250 and 260 °C, respectively. The oven was held at 100 °C for 5 min, raised to 180 °C at 8 °C/min and held for 10 min, and then raised to 220 °C at 2 °C/min and held for 15 min. Methyl esters were identified against a certified 37-component standard mixture (Supelco 37 Component FAME Mix, Sigma-Aldrich, St. Louis, MO, USA) and quantified as relative percentages of total fatty acid methyl esters. The composition is reported in Table S3.

### 2.3. Animals and Diets

All animal procedures adhered to the ethical guidelines for the use of live animals in scientific research (EU Directive 2010/63/EU [28]) and to Algerian Law no. 88-08 of 26 January 1988 on veterinary medical activities and animal health protection, as amended. The experimental protocol was approved by the Institutional Animal Ethics Committee of the University of Oran 1 Ahmed Ben Bella (registration no. 06/2021). The 3Rs principle was applied to minimize the number of animals used and their discomfort, and reporting follows the ARRIVE 2.0 guidelines.

In total, 30 male Wistar rats (Pasteur Institute, Algiers, Algeria), weighing 180 ± 20 g, were used. The animals were conventional, non-genetically modified outbred Wistar rats, naïve to any previous experimental procedure. After 1 week of acclimatization, rats were ranked by initial body weight and randomly divided into three groups (*n* = 10 per group) stratified by body weight, so that mean initial body weight did not differ between groups: a control group (C) fed a diet containing 20% casein; a high-fat diet group (HFD) fed a diet containing 20% casein enriched with 20% sheep fat; and a high-fat diet group in which casein was replaced by 20% chickpea protein isolate (HFD-CP). Diets were administered for 4 weeks (28 days). Diets were supplemented with AIN-93 mineral mix (AIN-93M-MX, 40 g/kg) and AIN-93 vitamin mix (AIN-93-VX, 20 g/kg), both obtained from MP Biomedicals (Solon, OH, USA). The detailed composition of the experimental diets is provided in Table S1. No a priori sample size calculation was performed; the group size of 10 animals was based on previous studies conducted in our laboratory using comparable high-fat-diet rat models and on the 3Rs principle of using the minimum number of animals required to obtain interpretable results. A sensitivity analysis indicated that, with 10 animals per group, a two-sided α of 0.05, and 80% power, the study was able to detect a standardized between-group difference of 1.32 standard deviations. This was an exploratory study; no single primary outcome measure was pre-specified, and all outcomes were analysed with equal weight. No inclusion or exclusion criteria were set a priori, and all animals were included in the analysis. All 30 animals completed the 28-day experimental period, with no animal loss, exclusion, or adverse event observed. To minimize bias, tissue sampling, biochemical assays, and outcome evaluations were performed blinded to group allocation.

Food and tap water were provided ad libitum. Ad libitum feeding was chosen deliberately because voluntary food intake is itself an outcome of interest when a novel protein source is introduced into a high-fat diet and because pair feeding would have concealed any effect of the isolate on intake; the consequence for the interpretation of the growth data is considered in the Discussion. Animals were kept under controlled conditions (23 ± 1 °C, 60% relative humidity, 12 h light/dark cycle). Rats were housed individually in polypropylene cages (42 cm × 26 cm × 18 cm) with autoclaved, dust-free wood chip bedding changed twice weekly, which allowed body weight to be recorded weekly and food intake to be measured daily for each animal. Animals were inspected daily for signs of ill health or distress throughout the study. Environmental enrichment, consisting of wooden chew blocks and cardboard nesting tunnels, was provided in each cage in accordance with the ARRIVE 2.0 housing and husbandry recommendations. Energy density was calculated using Atwater factors (17 kJ/g for protein and carbohydrate, 37 kJ/g for fat), and daily energy intake was calculated as food intake (g/day) multiplied by the energy density of the corresponding diet. The food efficiency ratio (FER) was calculated individually for each rat as [daily body weight gain (g/day)/daily food intake (g/day)] × 100, and organ relative weight (RW) as [organ weight (g)/body weight (g)] × 100. Group values were summarized as mean, standard deviation, and standard error of the mean (SEM).

### 2.4. Sample Collection

At the end of the 28-day experimental period, following an overnight fast (12 h), rats were anesthetized by intraperitoneal injection of a 10% chloral hydrate solution (3 mL/kg body weight). Chloral hydrate was selected to avoid volatile halogenated anesthetics that can alter systemic lipid metabolism and hepatic enzyme activities and was the injectable agent routinely validated for terminal procedures in our laboratory. Surgical depth of anesthesia was systematically confirmed prior to blood sampling by the loss of the pedal withdrawal reflex, absence of the corneal reflex, and unresponsiveness to noxious mechanical stimuli. Blood was collected by abdominal aorta exsanguination, which served as the primary humane method of euthanasia under deep surgical anesthesia; death was subsequently confirmed by cervical dislocation, in full compliance with animal welfare guidelines for adult rats [28]. Serum was obtained by low-speed centrifugation (3000 rpm, approximately 1500 x *g*, 20 min, 4 °C). Fresh serum was used immediately for LCAT and PON-1 activity assays; 0.1% (*w*/*v*) EDTA-Na_2_ was added to the remaining serum aliquots. Liver and epididymal adipose tissue were rapidly excised, rinsed in ice-cold physiological saline (0.9% NaCl), blotted dry, weighed, and stored at −70 °C until analysis.

### 2.5. Serum Leptin and Lipid Analyses

Serum leptin was measured by radioimmunoassay (Assay Designs Inc., Ann Arbor, MI, USA; cat. no. 900-028). Liver lipids were extracted according to Folch et al. [24]. Serum total cholesterol (TC) and triglycerides (TG) were determined enzymatically using commercial kits (TC: Biocon Diagnostics, Vohl-Marienhagen, Germany; TG: GPO-POD, Spinreact, Sant Esteve de Bas, Spain, cat. no. 1001311). HDL-cholesterol (HDL-C) and LDL-cholesterol (LDL-C) were measured directly using homogeneous enzymatic colorimetric assays (both Biocon Diagnostics, Vohl-Marienhagen, Germany). Serum lipid concentrations were expressed in mmol/L and hepatic TG and cholesterol contents in mg/g of wet liver tissue.

### 2.6. Determination of Oxidative Stress Markers

Lipid peroxidation in serum and liver was assessed as thiobarbituric acid reactive substances (TBARS), expressed as malondialdehyde equivalents, in micromoles per litre for serum and in nanomoles per milligram of protein for liver, according to Barber et al. [29]. Superoxide dismutase (SOD), catalase (CAT), and glutathione peroxidase (GPx) activities were measured using commercial kits (Cayman Chemical, Ann Arbor, MI, USA). Protein concentrations in liver homogenates were determined by the method of Lowry et al. [30].

### 2.7. Determination of LCAT and PON-1 Activities

LCAT activity was determined on fresh serum by an endogenous method [31], measuring the disappearance of unesterified cholesterol between two time points 4 h apart with a colorimetric enzymatic kit (CHOD-PAP, Biolabo, Maizy, France), and expressed in nmol of esterified cholesterol/mL/h. PON-1 activity was estimated by the method of La Du et al. [22], based on the hydrolysis of phenyl acetate to phenol; the arylesterase activity was calculated using the molar absorption coefficient of phenol (1310 M^−1^ cm^−1^), one unit corresponding to 1 µmol of phenol formed per minute.

### 2.8. Statistical Analysis

Data are expressed as mean ± standard error of the mean (SEM) for 10 rats per group (*n* = 10). Prior to inferential statistical evaluation, the normality of data distribution was verified using the Shapiro–Wilk test, and the homogeneity of variances was confirmed using Levene’s test; both parametric assumptions were met for all analyzed variables (*p* > 0.05). Statistical comparisons among experimental groups were conducted using one-way analysis of variance (ANOVA), followed by Tukey’s honestly significant difference (HSD) post hoc test for multiple pairwise comparisons [32]. Means with different superscript letters differ significantly at *p* < 0.05. Statistical analyses were performed using GraphPad Prism software (version 9.5.1, GraphPad Software Inc., San Diego, CA, USA).

## 3. Results

### 3.1. Chemical and Amino Acid Composition of the Isolate

Protein purity was 96% for casein and approximately 93% for the chickpea protein isolate (Table 1). Compared with casein, the chickpea protein isolate showed a distinct amino acid profile, with a markedly higher content of arginine, aspartic acid, and glycine. In contrast, methionine, leucine, and valine were lower than in casein, while isoleucine was comparable. The lysine-to-arginine ratio was markedly reduced in the chickpea protein isolate. The higher arginine content and lower lysine-to-arginine ratio are consistent with the hypocholesterolemic potential attributed to pulse proteins.

Protein quality indices derived from this profile are given in Table 2. None of the eight indispensable amino acids determined fell below the FAO/WHO reference pattern for the older child, adolescent, and adult, in either protein. In the chickpea protein isolate, valine was the first limiting amino acid, giving an amino acid score of 115%, while the lysine ratio reached 142%, in contrast with the lysine deficiency that characterizes most plant proteins. Against the more demanding pattern for the child aged 6 months to 3 years, the sulfur amino acids became first limiting, with a score of 104%. Casein reached higher scores (139% and 119%, sulfur amino acids limiting against both patterns) and a higher indispensable amino acid content (44.3 versus 35.8 g/100 g protein, corresponding to indispensable to dispensable amino acid ratios of 0.78 and 0.62). The lysine-to-arginine ratio was 0.81 in the chickpea protein isolate against 2.12 in casein.

### 3.2. Body Weight, Food Intake, and Relative Organ Weights

After 28 days, HFD rats showed increased final body weight, body weight gain, food and energy intake, FER, and relative liver and adipose tissue weights compared with controls (Table 3). Compared with HFD, HFD-CP rats showed a lower body weight gain (−19%) and a lower relative epididymal adipose tissue weight (−23%) (*p* < 0.05). Final body weight, food intake, energy intake, FER, and relative liver weight were all numerically lower in HFD-CP than in HFD rats, by 11%, 14%, 14%, 5%, and 13%, respectively, but none of these differences reached significance. Food intake and relative liver weight in HFD-CP rats were intermediate and did not differ significantly from either of the two other groups, whereas final body weight and energy intake remained significantly higher than in controls.

### 3.3. Serum Leptin, Lipoproteins, and Liver Lipids

HFD feeding markedly increased serum leptin, total cholesterol, triglycerides, and LDL-cholesterol and decreased HDL-cholesterol (Table 4). Compared with HFD, replacing casein with chickpea protein isolate reduced serum leptin (−29%), total cholesterol (−23%), LDL-cholesterol (−36%), hepatic triglycerides (−26%), and hepatic cholesterol (−29%) (*p* < 0.05). Serum triglycerides were 21% lower and HDL-cholesterol 31% higher than in HFD rats, but neither difference reached significance. Serum leptin, total cholesterol, triglycerides, and hepatic cholesterol in HFD-CP rats no longer differed significantly from control values, whereas LDL-cholesterol and hepatic triglycerides remained higher than in controls.

### 3.4. Oxidative Stress Markers

Serum and hepatic TBARS were significantly higher in HFD rats than in controls (Table 5). Compared with HFD, replacing casein with chickpea protein isolate reduced serum TBARS (−29%) and hepatic TBARS (−35%) and increased SOD (+41%) and CAT (+36%) activities (*p* < 0.05). GPx activity was 36% higher in HFD-CP than in HFD rats, but the difference did not reach significance. Serum TBARS, SOD, and CAT values in HFD-CP rats did not differ significantly from those of controls, whereas hepatic TBARS remained higher than in controls.

### 3.5. LCAT and PON-1 Activities

LCAT and PON-1 activities were markedly reduced in HFD rats compared with controls (−38% and −36%, respectively) (Table 6). Replacing casein with chickpea protein isolate increased LCAT (+37%) and PON-1 (+33%) activities compared with HFD (*p* < 0.05), restoring both to values that no longer differed significantly from those of control rats.

## 4. Discussion

Substituting a chickpea protein isolate for casein markedly improved the metabolic alterations induced by high-fat diet (HFD) feeding. The isolate reduced body weight gain and adiposity, attenuated hepatic lipid accumulation, improved the serum lipid profile, decreased oxidative stress, and enhanced HDL-associated enzymatic activities (LCAT and PON-1), suggesting coordinated effects on interconnected pathways linking dyslipidemia, oxidative stress, and hepatic lipid accumulation. Read as a quality assessment of the ingredient, these results indicate that the protein fraction extracted from chickpea grain retains and, in this dietary context, improves upon the nutritional performance of a reference animal protein.

Consistent with established HFD models, obesogenic-diet rats exhibited increased body weight gain; elevated FER; expanded adipose mass, hyperleptinemia, and dyslipidemia; and increased hepatic triglyceride and cholesterol content, hallmarks of diet-induced metabolic dysfunction and early-stage NAFLD [33,34]. Chickpea protein reduced body weight gain (−19%) and adipose tissue relative weight (−23%). Energy intake was 14% lower in HFD-CP than in HFD rats; although this difference did not reach statistical significance, a contribution of lower voluntary intake to the observed metabolic changes cannot be excluded. Recalculated on an as-fed basis, the two high-fat diets remained isoenergetic, 18.97 against 18.96 MJ/kg, a difference of 0.07%, because the small protein deficit of the isolate is almost exactly offset by its additional residual lipid. They were matched for added sheep fat and differed in the protein ingredient, in a protein content of 186.4 against 192.0 g/kg and in 2.6 g/kg of additional non-protein solids carried by the isolate, so a difference in added sheep fat does not explain the findings. The increases in LCAT, PON-1, and antioxidant enzyme activities, moreover, represent qualitative changes in lipoprotein function and redox status that are less readily explained by differences in energy intake alone. Taken together, these observations are compatible with a contribution of the chickpea protein isolate itself, but the present design cannot establish it for body weight and adiposity. The food efficiency ratio did not differ between the two high-fat groups, so the lower weight gain of HFD-CP rats is consistent with a modest reduction in voluntary intake that the study was not powered to detect. The ad libitum design cannot distinguish effects of the isolate itself from effects mediated by altered voluntary intake, by satiation or palatability, or by the difference in sulfur amino acid supply. A pair-fed study, matched for nitrogen and for sulfur amino acids, is required to resolve this question.

The hyperleptinemia in HFD rats is consistent with adipose expansion and possibly leptin resistance, a common feature of obesity [35]. The 29% reduction in serum leptin in HFD-CP rats parallels the decrease in adiposity. Because leptin resistance is closely associated with hepatic lipid accumulation [36,37], this reduction may have contributed to the attenuation of hepatic lipid accumulation in the chickpea protein group.

A central finding is the marked reduction in hepatic triglyceride and cholesterol content following the replacement of casein with chickpea protein isolate. Hepatic lipid accumulation under high-fat feeding results from enhanced de novo lipogenesis and impaired fatty acid oxidation [17]. Although hepatic gene expression was not assessed, the concomitant decrease in serum and hepatic lipids is consistent with a shift away from lipid storage toward improved lipid handling, possibly related to the amino acid composition of chickpea protein and to bioactive peptides released during digestion.

The hypolipidemic effects further support a beneficial modulation of lipid metabolism. Chickpea protein significantly reduced total cholesterol and LDL-cholesterol and shifted serum triglycerides and HDL-cholesterol in a favourable direction without reaching significance, indicating a less atherogenic profile. The distinctive amino acid composition of chickpea protein, with elevated arginine and a reduced lysine-to-arginine ratio, may partly explain these effects, as lower lysine-to-arginine ratios have been associated with improved cholesterol metabolism [6,11]. The amino acid ratios reported in Table 2 further indicate that this favourable arginine profile is not obtained at the expense of the overall indispensable amino acid supply, since no indispensable amino acid determined fell below the FAO/WHO reference pattern. The higher arginine content and the lower lysine-to-arginine ratio of the isolate offer a plausible, but in the present study untested, explanation for the changes observed in LCAT and PON-1 activity. Plasma arginine, the plasma lysine-to-arginine ratio, nitrite and nitrate concentrations, and vascular function were not measured here, and this interpretation must therefore be regarded as a hypothesis to be tested directly rather than as a mechanism established by the present data.

Beyond quantitative changes in HDL-cholesterol, chickpea protein improved two enzymatic determinants of HDL functionality. LCAT activity, reduced by HFD, increased by 37%; LCAT is essential for reverse cholesterol transport, esterifying free cholesterol and promoting HDL maturation [38,39]. PON-1 activity, which declined under HFD, increased by 33%; PON-1 protects lipoproteins from oxidative modification and maintains HDL antioxidant function [22,40]. That LCAT and PON-1 increased while HDL-cholesterol concentration did not change significantly suggests that the improvement concerned HDL quality rather than HDL quantity, which is precisely the distinction that measurements of HDL-cholesterol alone cannot capture.

Oxidative stress is a key mediator linking dyslipidemia, hepatic lipid accumulation, and atherosclerotic risk [20,40]. HFD feeding increased serum and hepatic TBARS and decreased SOD, CAT, and GPx activities. Chickpea protein reduced TBARS and increased SOD and CAT activities, whereas the increase in GPx activity did not reach significance. Improved redox balance likely contributed to the preservation of HDL functionality, since oxidative stress inactivates HDL-associated enzymes [21,41]. The antioxidant effect of chickpea protein may be attributed to its amino acid profile and to bioactive peptides generated during digestion, which exert radical-scavenging properties [6,9,42]. The simultaneous improvement in lipid metabolism, hepatic lipid content, oxidative stress, and HDL enzymatic activities suggests an integrated metabolic response, in which the isolate appears to act on several nodes of an interdependent network rather than on a single pathway.

Unlike previous studies focusing on whole pulses or crude protein fractions, the present work evaluated a purified chickpea protein isolate, which reduces, but does not eliminate, the contribution of fibre and other seed constituents to the observed effects. This distinction matters for food formulation: it is the isolated protein fraction, not the whole seed, that is incorporated into products, and its nutritional performance is therefore a quality attribute of the fractionation process as much as of the grain. Because the isolate was prepared by alkaline solubilization and isoelectric precipitation, a route that is accessible to small and medium processing units in producing regions, the results also suggest that local fractionation of chickpea grain could generate a high-value ingredient without recourse to capital-intensive membrane technology. Confirming this will require the effects reported here to be reproduced across cultivars, growing seasons, and extraction conditions, since seed composition and processing parameters both influence the final amino acid and peptide profile [9].

A potential confounder deserves explicit consideration. The chickpea protein isolate provided less methionine than casein (1.3 versus 2.4 g/100 g protein, corresponding to 2.42 and 4.61 g/kg diet, respectively), and dietary methionine restriction is itself capable of reducing body weight gain, hepatic lipid content, and markers of oxidative stress in rodents. A contribution of this difference to the present findings, therefore, cannot be dismissed a priori. Several considerations nevertheless argue against methionine restriction as the principal explanation. First, methionine intake in the HFD-CP group remained well above the threshold below which cystine can no longer spare methionine in the growing rat, and the sulfur amino acid supply of this diet is therefore better expressed as total methionine plus cystine than as methionine alone. The chickpea protein isolate was markedly richer in cystine than casein (1.5 versus 0.8 g/100 g protein), so that, expressed as methionine equivalents and calculated on an as-fed basis from the actual protein content of the diets, the chickpea-based diet provided 4.68 g/kg and the casein-based diets 5.85 g/kg. The difference in sulfur amino acid supply was thus 20%, rather than the 47% apparent for methionine alone. The HFD-CP diet, therefore, fell approximately 4.5% below the estimated sulfur amino acid requirement of the growing rat of about 4.9 g methionine equivalents/kg diet, and a mild sulfur amino acid insufficiency in that group cannot be excluded. The estimated shortfall is small in absolute terms, 0.22 g/kg, but its biological relevance cannot be excluded and would require direct testing in a nitrogen- and sulfur-amino-acid-matched design. Second, whether a modest reduction in sulfur amino acid supply contributes to the observed changes in LCAT and PON-1 activity remains uncertain. The present experiment was not designed to isolate effects of sulfur amino acid supply, and we are not aware of published evidence bearing directly on the question; absence of such evidence is not evidence of absence. We, therefore, attribute the observed effects to the isolate as an ingredient rather than to its protein fraction alone. Third, the amino acid profile of chickpea protein offers an independent and well-documented basis for the lipid-lowering effects observed, in particular, its high arginine content and correspondingly low lysine-to-arginine ratio [7]. We nonetheless acknowledge that only a study using methionine-matched diets can definitively disentangle these contributions.

This study has limitations. The sample size allowed the detection of large between-group differences only, and smaller but biologically relevant effects may therefore have gone undetected. A single cultivar, growing site, and extraction protocol were used, so cultivar and processing effects on the nutritional performance of the isolate remain unknown. Insulin sensitivity, inflammatory cytokines, intestinal permeability, and short-chain fatty acid production were not assessed, protein digestibility was not measured, and hepatic regulatory pathways were not examined at the molecular level. Hepatic lipid accumulation was evaluated biochemically rather than histologically. Future studies combining metabolic phenotyping with molecular and histological analyses and extending the comparison to several cultivars and extraction routes are warranted to confirm the underlying mechanisms and their robustness across ingredient batches. Several further limitations should be stated. The proximate fractions determined for both protein sources sum to 100%, so no carbohydrate fraction was estimated by difference, and residual oligosaccharides and soluble fibre in the isolate were not determined. The HFD-CP diet fell marginally below the cited sulfur amino acid requirement, and methionine supplementation, with matching of the diets on a nitrogen basis, is required in future work. Cholesterol was not determined in the diets or in the fat ingredient, and choline concentrations were not determined in the finished diets. Neither diet contained added vegetable oil, so the ovine fat was the sole source of essential fatty acids in the two high-fat diets, and the control diet contained very little; this qualifies comparisons with the control group but not the comparison between HFD and HFD-CP. Plasma arginine, the plasma lysine-to-arginine ratio, nitrite and nitrate concentrations, and vascular function were not measured.

## 5. Conclusions

A chickpea (*Cicer arietinum* L.) protein isolate prepared from locally produced grain attenuated hepatic lipid accumulation and dyslipidemia in high-fat-diet-fed rats. Beyond reducing serum and hepatic lipids, it restored antioxidant defences and enhanced HDL functionality through increased LCAT and paraoxonase-1 activities, without a significant change in HDL-cholesterol concentration. Because these effects were obtained when the isolate replaced casein in diets containing the same amount of added sheep fat, they are attributable to the isolate as an ingredient rather than to the fibre and phytochemicals of the whole seed. The isolate also carries a small residual lipid and mineral fraction and was not matched to casein on a nitrogen basis, so the present design does not allow the effects to be assigned to the protein moiety in isolation. The nutritional quality of chickpea protein isolate can therefore be regarded as a quality attribute of the ingredient itself, and its documentation supports the use of chickpea protein isolates in the formulation of plant-based foods.

## Figures and Tables

**Table 1:** Chemical and amino acid composition (%) of chickpea protein isolate (CP) and casein.

Component	CP	Casein
***Chemical composition***		
Crude protein (%)	93.2 ± 0.4	96.0 ± 0.3
Crude fat (%)	1.4 ± 0.1	0.3 ± 0.0
Ash (%)	2.6 ± 0.1	2.4 ± 0.1
Moisture (%)	2.8 ± 0.2	1.3 ± 0.1
***Amino acid composition (g/100 g protein)***		
Alanine	3.9 ± 0.1	2.9 ± 0.1
Arginine	8.4 ± 0.2	3.4 ± 0.1
Aspartic acid	10.8 ± 0.3	6.2 ± 0.1
Glutamic acid	16.9 ± 0.4	19.8 ± 0.3
Glycine	4.1 ± 0.1	1.7 ± 0.1
Histidine	2.6 ± 0.1	5.2 ± 0.1
Isoleucine	4.4 ± 0.1	4.9 ± 0.1
Leucine	7.3 ± 0.2	9.2 ± 0.2
Lysine	6.8 ± 0.2	7.2 ± 0.1
Methionine	1.3 ± 0.1	2.4 ± 0.1
Cystine	1.50 ± 0.08	0.80 ± 0.01
Total sulfur amino acids (Met + Cys)	2.8	3.2
Phenylalanine	5.1 ± 0.2	4.8 ± 0.1
Proline	4.2 ± 0.2	11.5 ± 0.3
Serine	4.8 ± 0.1	5.4 ± 0.1
Threonine	3.7 ± 0.1	4.7 ± 0.1
Tyrosine	3.3 ± 0.1	5.2 ± 0.1
Valine	4.6 ± 0.1	5.9 ± 0.1
Lysine/arginine ratio	0.81	2.12
Methionine/glycine ratio	0.32	1.41

Values are means ± SEM of triplicate analyses. Cystine was determined after performic acid oxidation. Tryptophan was not determined because it is destroyed by acid hydrolysis. CP: chickpea protein isolate.

**Table 2:** Amino acid ratios and protein-quality indices of chickpea protein isolate (CP) and casein, calculated against the FAO/WHO 2013 reference patterns.

Indispensable Amino Acid	Reference (mg/g)	CP	Casein
Histidine	16	163	325
Isoleucine	30	147	163
Leucine	61	120	151
Lysine	48	142	150
Sulfur amino acids (Met + Cys)	23	122	139
Aromatic amino acids (Phe + Tyr)	41	205	244
Threonine	25	148	188
Valine	40	115	148
***Protein quality indices***			
Amino acid score (%), older child to adult	–	115	139
First limiting amino acid, older child to adult	–	Valine	Met + Cys
Amino acid score (%), child 6 months to 3 years	–	104	119
First limiting amino acid, child 6 months to 3 years	–	Met + Cys	Met + Cys
Essential amino acid index (%)	–	143	181
Indispensable amino acids (g/100 g protein)	–	35.8	44.3
Indispensable to dispensable amino acid ratio	–	0.62	0.78
Indispensable amino acids (% of total)	–	38.2	43.8
Lysine-to-arginine ratio	–	0.81	2.12

Values in the CP and casein columns are amino acid ratios, expressed as a percentage of the corresponding FAO/WHO 2013 reference pattern for the older child, adolescent, and adult [27]. The amino acid score is the lowest of the individual ratios and identifies the first limiting amino acid. The essential amino acid index is the geometric mean of the individual ratios. Tryptophan was not determined, so all indices are based on eight of the nine indispensable amino acids. Indices are not corrected for digestibility and are consequently neither PDCAAS nor DIAAS values. The indispensable amino acid content is the sum of the eight indispensable amino acids determined; cystine and tyrosine are included only in the sulfur and aromatic amino acid ratios, respectively.

**Table 3:** Growth parameters and organ relative weight (RW).

Parameter	C	HFD	HFD-CP
***Growth parameters***			
Initial body weight (g)	172 ± 8	173 ± 9	171 ± 7
Final body weight (g)	298 ± 12 ^b^	412 ± 18 ^a^	365 ± 15 ^a^
Body weight gain (g/day)	4.5 ± 0.3 ^c^	8.5 ± 0.4 ^a^	6.9 ± 0.3 ^b^
Food intake (g/day)	19.2 ± 1.1 ^b^	23.8 ± 1.3 ^a^	20.4 ± 1.0 ^ab^
Energy intake (kJ/day)	286 ± 16 ^b^	455 ± 25 ^a^	390 ± 19 ^a^
FER (%)	23.4 ± 2.1 ^b^	35.9 ± 2.6 ^a^	34.0 ± 2.2 ^a^
***Relative organ weight (%)***			
Liver	2.48 ± 0.12 ^b^	3.21 ± 0.15 ^a^	2.79 ± 0.14 ^ab^
Epididymal adipose tissue	2.12 ± 0.10 ^c^	3.54 ± 0.18 ^a^	2.71 ± 0.16 ^b^

Data are expressed as mean ± SEM for 10 rats per group. C: control diet; HFD: high-fat diet; HFD-CP: high-fat diet in which casein was replaced by 20% chickpea protein isolate. Data were analyzed by one-way ANOVA followed by Tukey’s HSD post hoc test; means with different superscript letters are significantly different (*p* < 0.05). FER: food efficiency ratio.

**Table 4:** Serum leptin, serum lipid profile, and hepatic lipid concentrations.

Parameter	C	HFD	HFD-CP
Serum leptin (ng/mL)	2.9 ± 0.3 ^b^	5.8 ± 0.5 ^a^	4.1 ± 0.4 ^b^
***Serum lipids (mmol/L)***			
Total cholesterol	1.45 ± 0.12 ^b^	2.36 ± 0.18 ^a^	1.82 ± 0.15 ^b^
Triglycerides	0.78 ± 0.06 ^b^	1.21 ± 0.09 ^a^	0.95 ± 0.07 ^ab^
HDL-cholesterol	0.72 ± 0.05 ^a^	0.48 ± 0.04 ^b^	0.63 ± 0.05 ^ab^
LDL-cholesterol	0.41 ± 0.04 ^c^	1.18 ± 0.10 ^a^	0.76 ± 0.08 ^b^
***Hepatic lipids***			
Hepatic triglycerides (mg/g wet liver)	18.5 ± 1.6 ^c^	36.2 ± 2.4 ^a^	26.8 ± 2.1 ^b^
Hepatic total cholesterol (mg/g wet liver)	14.7 ± 1.2 ^b^	28.9 ± 2.0 ^a^	20.4 ± 1.8 ^b^

Data are expressed as mean ± SEM for 10 rats per group. Group abbreviations and statistical analysis, as in Table 3. Means in a row with different superscript letters are significantly different (*p* < 0.05).

**Table 5:** Oxidative stress markers in serum and liver.

Parameter	C	HFD	HFD-CP
***Serum***			
TBARS (µmol/L)	3.8 ± 0.3 ^b^	7.2 ± 0.6 ^a^	5.1 ± 0.4 ^b^
***Liver***			
TBARS (nmol/mg protein)	1.9 ± 0.2 ^c^	4.6 ± 0.4 ^a^	3.0 ± 0.3 ^b^
SOD (U/mg protein)	8.7 ± 0.6 ^a^	5.1 ± 0.4 ^b^	7.2 ± 0.5 ^a^
CAT (U/mg protein)	62.4 ± 4.2 ^a^	38.7 ± 3.1 ^b^	52.6 ± 3.8 ^a^
GPx (U/mg protein)	12.3 ± 0.9 ^a^	7.4 ± 0.6 ^b^	10.1 ± 0.8 ^ab^

Data are expressed as mean ± SEM for 10 rats per group. Group abbreviations and statistical analysis, as in Table 3. Means in a row with different superscript letters are significantly different (*p* < 0.05). TBARS are expressed as malondialdehyde equivalents. TBARS: thiobarbituric acid reactive substances; SOD: superoxide dismutase; CAT: catalase; GPx: glutathione peroxidase.

**Table 6:** LCAT and paraoxonase-1 (PON-1) activities.

Parameter	C	HFD	HFD-CP
LCAT activity (nmol/mL/h)	62.4 ± 4.1 ^a^	38.6 ± 3.2 ^b^	52.8 ± 3.7 ^a^
PON-1 activity (U/mL)	118.5 ± 7.8 ^a^	76.2 ± 6.1 ^b^	101.3 ± 7.0 ^a^

Data are expressed as mean ± SEM for 10 rats per group. Group abbreviations and statistical analysis, as in Table 3. Means in a row with different superscript letters are significantly different (*p* < 0.05). LCAT: lecithin-cholesterol acyltransferase.

## Data Availability

The original contributions presented in this study are included in the article/Supplementary Material. Further inquiries can be directed to the corresponding author.

## Supplementary Materials

The following supporting information can be downloaded at: https://www.mdpi.com/article/10.3390/foods15203765/s1, Table S1: Composition of the experimental diets (g/kg); Table S2: Calculated as-fed composition of the experimental diets (g/kg); Table S3: Fatty acid composition of the sheep fat used in the experimental diets and calculated fatty acid content of the three diets.
