# Supplementary material for: Chickpea (Cicer arietinum L.) Protein Isolate: Amino Acid Quality and Enhanced HDL Functionality in High-Fat-Diet-Fed Rats
Source: Foods. 2026 Oct 21;15(20):3765. doi: 10.3390/foods15203765 (PMC13704737; doi:10.3390/foods15203765)
Supplement: Supplementary file 1 [file foods-15-03765-s001.zip › foods-4541018-supplementary.pdf]

Supplementary Material

Table S1. Composition of the experimental diets (g/kg).

Ingredient	C	HFD	HFD-CP
Casein	200	200	–
Chickpea protein isolate	–	–	200
Corn starch	647.5	447.5	447.5
Sucrose	40	40	40
Sheep fat	–	200	200
Cellulose	50	50	50
Mineral mix (AIN-93M-MX)	40	40	40
Vitamin mix (AIN-93-VX)	20	20	20
Choline bitartrate	2.5	2.5	2.5
Total	1000	1000	1000

Diets were given in powdered form. Calculated energy density (Atwater factors): C, 15.0 MJ/kg; HFD, 19.0 MJ/kg; HFD-CP, 19.0 MJ/kg (39% of energy from fat). HFD and HFD-CP were isoenergetic, the calculated difference in energy density being 0.07%, and were matched for added sheep fat. They differed in the protein ingredient and therefore also in the small residual lipid, mineral, moisture and amino acid contributions of the respective protein sources. The diets are modified AIN-93M formulations. C: control diet; HFD: high-fat diet; HFD-CP: high-fat diet in which casein was replaced by 20% chickpea protein isolate. The mineral mix (AIN-93M-MX, 40 g/kg) and the vitamin mix (AIN-93-VX, 20 g/kg) were obtained from MP Biomedicals (Solon, OH, USA).

Table S2. Calculated as-fed composition of the experimental diets (g/kg).

Component	C	HFD	HFD-CP
Protein from the protein source	192.0	192.0	186.4
Lipid from the protein source	0.6	0.6	2.8
Sheep fat	–	200	200
Total lipid	0.6	200.6	202.8
Ash from the protein source	4.8	4.8	5.2
Moisture from the protein source	2.6	2.6	5.6
Cellulose (added fibre)	50	50	50
Methionine	4.61	4.61	2.42
Methionine + cystine	6.14	6.14	5.22
Sulfur amino acids as methionine equivalents	5.85	5.85	4.68
Energy density (MJ/kg)	15.0	19.0	19.0

Values were calculated from the inclusion rate of each protein source in Table S1 and from its measured composition in Table 1. Sulfur amino acids are expressed as methionine equivalents, 1.24 g cystine

being taken as equivalent to 1.0 g methionine. Energy density was calculated with Atwater factors. The proximate fractions determined for both protein sources sum to 100%, so no carbohydrate fraction was estimated by difference; residual oligosaccharides and soluble fibre in the isolate were not determined.

Table S3. Fatty acid composition of the sheep fat used in the experimental diets, and calculated fatty acid content of the three diets.

(a) Sheep fat, batch SF-2024-06 (g/100 g total fatty acids, mean ± SD, n = 3).

**Fatty acid**	**Common name**	**g/100 g**
C14:0	Myristic	3.8 ± 0.2
C16:0	Palmitic	24.5 ± 0.8
C17:0	Margaric	1.8 ± 0.1
C18:0	Stearic	23.2 ± 0.7
C10:0, C12:0, C15:0	Minor saturated	1.2
Total saturated		54.5
C16:1 n-7	Palmitoleic	2.4 ± 0.1
C18:1 n-9	Oleic	34.6 ± 0.9
C18:1 t11	Vaccenic	1.8 ± 0.1
Total monounsaturated		38.8
C18:2 n-6	Linoleic	2.8 ± 0.1
C18:3 n-3	α-Linolenic	0.9 ± 0.05
C18:2 c9,t11	Rumenic (CLA)	0.5 ± 0.03
Total polyunsaturated		4.2
Unidentified		2.5

(b) Calculated fatty acid content of the diets (g/kg diet).

	**C**	**HFD**	**HFD-CP**
Total lipid	0.6	200.6	202.8
Saturated	0.4	109.4	109.4
Monounsaturated	0.2	77.8	78.3
Linoleic acid (C18:2 n-6)	0.01	5.6	7.1
α-Linolenic acid (C18:3 n-3)	0.00	1.8	1.9
Rumenic acid (CLA)	–	1.0	1.0
Saturated, % of total fatty acids	65	54.5	53.9

Panel (a) reports the composition of the sheep fat batch used in the diets, determined by GC-FID as described in Section 2.2. Panel (b) was calculated from that measured composition and from the ingredient inclusion rates in Table S1. The fatty acid contributions of the residual lipid of the protein sources, 0.6 g/kg from casein and 2.8 g/kg from the chickpea protein isolate, were estimated from published milk fat and chickpea seed oil profiles respectively; the values in panel (b) should therefore be read as calculated estimates and not as direct measurements of the finished diets. Cholesterol was not determined. Because the same nominal amount of sheep fat was added to HFD and

HFD-CP, its cholesterol contribution was intended to be comparable between the two high fat diets; this was not analytically verified.
